# Distinct Clinicopathological Patterns of Mismatch Repair Status in Colorectal Cancer Stratified by *KRAS* Mutations

**DOI:** 10.1371/journal.pone.0128202

**Published:** 2015-06-04

**Authors:** Wenbin Li, Wenxue Zhi, Shuangmei Zou, Tian Qiu, Yun Ling, Ling Shan, Susheng Shi, Jianming Ying

**Affiliations:** Department of pathology, Cancer Hospital, Chinese Academy of Medical Sciences & Peking Union Medical College, Beijing, China; Sapporo Medical University, JAPAN

## Abstract

In sporadic colorectal cancer (CRC), the *BRAF^V600E^* mutation is associated with deficient mismatch repair (MMR) status and inversely associated with to KRAS mutations. In contrast to deficient MMR (dMMR) CRC, data on the presence of *KRAS* oncogenic mutations in proficient MMR (pMMR) CRC and their relationship with tumor progression are scarce. We therefore examined the MMR status in combination with *KRAS* mutations in 913 Chinese patients and correlated the findings obtained with clinical and pathological features. The MMR status was determined based on detection of MLH1, MSH2, MSH6 and PMS2 expression. *KRAS* mutation and dMMR status were detected in 36.9% and 7.5% of cases, respectively. Four subtypes were determined by MMR and *KRAS* mutation status: *KRAS* (+)/pMMR (34.0%), *KRAS* (+)/dMMR (2.9%), *KRAS* (-)/pMMR (58.5%) and *KRAS* (-)/dMMR (4.6%). A higher percentage of pMMR tumors with *KRAS* mutation were most likely to be female (49.0%), proximal located (45.5%), a mucinous histology (38.4%), and to have increased lymph node metastasis (60.3%), compared with pMMR tumors without *BRAF^V600E^* and *KRAS* mutations (36.0%, 29.3%, 29.4% and 50.7%, respectively; all *P* < 0.01). To the contrary, compared with those with *KRAS*(-)/dMMR tumors, patients with *KRAS*(+)/dMMR tumors demonstrated no statistically significant differences in gender, tumor location, pT depth of invasion, lymph node metastasis, pTNM stage, and histologic grade. This study revealed that specific epidemiologic and clinicopathologic characteristics are associated with MMR status stratified by *KRAS* mutation. Knowledge of MMR and *KRAS* mutation status may enhance molecular pathologic staging of CRC patients and metastatic progression in CRC can be estimated based on the combination of these biomarkers.

## Introduction

Colorectal cancer (CRC) is a heterogenous disease evolving from diverse genetic pathways and an accurate assessment of cancer based on tumor features would permit personalized cancer treatment [1,2,3]. Currently, anatomic and pathologic staging is still the most accurate predictor of patient outcome [4]. The discovery and validation of genetic markers determining the efficiency of metastatic progression of CRC is therefore an important area of research, with the potential value of defining the subset of patients at highest or lowest risk of relapse. One of the promising molecular markers investigated in CRC is the presence of tumor microsatellite instability (MSI) [5,6,7,8].

CRC is generally divided into two well-known molecular pathways, including the chromosomal instability (CIN) pathway and the microsatellite instability (MSI) pathway. MSI is the result of deficient DNA mismatch repair (dMMR) [9]. A germline mutation in one of the MMR genes, including *MLH1*, *MSH2*, *MSH6* or *PMS2*, is the cause of dMMR in patients with Lynch syndrome, which is an inherited disorder that increases the risk of developing CRC [10,11]. Deficient MMR is also observed in 10% to 20% of patients with sporadic CRC, of which the majority of dMMR tumors are due to hypermethylation of *MLH1* gene promoter, with *MSH2* and *MSH6* accounting for a smaller percentage [5]. Sporadic dMMR tumors, but not Lynch syndrome, frequently carry the activating somatic V600E mutation in the exon 15 of the *BRAF* oncogene [12,13,14]. Both sporadic and Lynch syndrome-associated tumors with dMMR status have distinct clinicopathologic features, such as preferential location in the proximal colon, prominent lymphocytic infiltrate, mucinous or signet ring differentiation, and association with a favorable prognosis. Data from the PETACC-3 trail reported that tumor specimens with dMMR status are more common in stage II disease than in stage III disease (22% *vs* 12%, *P* < 0.001) and with a percentage of 3.5% in stage IV tumors. These results indicate that dMMR tumors have a decreased likelihood to metastasize and suggest a more favorable outcome [9,15].

The Ras/Raf/MEK/ERK kinase cascade is involved in the control of cell proliferation, cell survival and invasion in CRC cancer cells [16]. *KRAS* is mutated in 35%-40% CRC and mutation of the *KRAS* protooncogene is an early event in development of these cancers, exerting a strong influence on the growth of colonic polyps and early cancers [17,18]. Robust evidence suggests the predictive value of *KRAS* mutation in metastatic CRC treated with anti-EGFR targeted therapy [19,20]. However, the clinical significance of *KRAS* mutation as a prognostic marker is controversial. Some studies reported no association with survival, whereas others suggested that patients with *KRAS* mutated tumors have poorer outcome for any mutation subtype [21,22,23,24].

The association of MMR status, *KRAS* and *BRAF* mutations on clinical outcome are frequently documented. However, development of a more accurate prediction on clinical outcome using biomarker combinations remains a worthy area of investigation. Furthermore, in contrast to dMMR CRC, data on the presence of *KRAS* oncogenic mutations in proficient MMR (pMMR) CRC and their relationship with tumor progression are scarce. The aim of this study was to evaluate the prognostic role of MMR status in combination with *KRAS* mutations in 913 Chinese patients and characterize the specified subtypes with respect to clinicopathologic features.

## Materials and Methods

### Study population

Patients with resected, histologically proven CRC were eligible. The clinicopathological records of 913 patients with corresponding paraffin-embedded materials available for molecular analysis were retrospectively collected from the Department of Pathology, Cancer Hospital, Chinese Academy of Medical Sciences, Beijing, China from 2011 to 2013. A central pathology review was performed. Stratification factors included: number of metastatic regional lymph nodes (N1: 1–3 *vs* N2: ≥4), histologic grade (G1-2: well/moderately differentiated *vs* G3: poorly differentiated/ undifferentiated), tumor diameter, pT classification, histological subtype, tumor location, tumor size as well as the pTNM stage. The pTNM staging system of the 7th edition AJCC cancer staging was used. Evaluation of M stage was mainly according to confirmed pathological results and/or radiological data. Proximal tumor site included cecum, ascending, hepatic flexure and transverse colon; distal site included splenic flexure, descending and sigmoid colon. Mucinous differentiation in the tumor was defined by the presence of pools of extracellular mucin-containing clusters of carcinomatous cells. When > 50% of analyzed tumor demonstrated mucinous differentiation, the tumor was classified as mucinous carcinoma. The study was approved by the Institute Review Board of the Cancer Hospital, Chinese Academy of Medical Sciences. Each participant signed an Institutional Review Board approved informed consent in accordance with current guidelines.

### *KRAS* and *BRAF* ^*V600E*^ mutation analysis

Assessment of *KRAS* and *BRAF* V600E mutational status was performed in the Molecular Pathology Laboratory of the Department of Pathology, Cancer Hospital, CAMS, using appropriate quality control procedures. Mutation status was determined using genomic DNA extracted from macrodissected formalin-fixed, paraffin-embedded tumor tissue. Both *KRAS* (codons 12 and 13) and *BRAF* (p.V600E) mutation tests were performed using a multiplex allele-specific PCR-based assay (ACCB, Beijing, China), together with the Stratagene Mx3000P (Agilent Technologies Inc, Santa Clara, CA), which assesses seven different potential mutations in *KRAS* codons 12 and 13 (*Gly12Ala*, *Gly12Asp*, *Gly12Arg*, *Gly12Cys*, *Gly12Ser*, *Gly12Val*, and *Gly13Asp*). Neither *KRAS* nor *BRAF* ^*V600E*^ mutated tumors were designated as wild-type *KRAS* subtype.

### DNA mismatch repair proteins expression

MMR protein (MLH1, PMS2, MSH2 and MSH6) expression was performed as a routine practice in our pathological department. All samples were stained in an autostainer (Autostainer Link 48, Dako, Denmark). Four μm thick tissue sections were deparaffinized in xylene, rehydrated in graded alcohol and washed in distilled water. Ready-to-use primary mouse monoclonal antibodies included MLH1 antibody (ES05, Dako) and MSH2 antibody (FE11, Dako). Ready-to-use primary rabbit monoclonal antibodies included MSH6 antibody (EP49, Dako) and PMS2 antibody (EP51, Dako). MMR protein loss was defined as absence of nuclear staining in tumor cells but positive nuclear staining in normal colonic epithelial cells and lymphocytes. Tumors were designated as dMMR status if loss of at least one MMR protein was detected and pMMR if all proteins were intact.

### *MLH1* promoter methylation analysis

All DNA specimens were subjected to bisulfite modification using the EZ DNA Methylation Kit (Zymo Research, CA, USA) according to the manufacturer instructions. One μg of genomic DNA from each sample was bisulfite converted and eluted in 18μl elution buffer. Methylation-specific PCR (MSP) was conducted as previously described [25]. The primers used for this analysis were: 5’-AAT TAA TAG GAA GAG CGG ATA GC-3’ and 5’-CCT CCC TAA AAC GAC TAC TAC CCG-3’ for methylated *MLH1* promoter and 5’-TGA ATT AAT AGG AAG AGT GGA TAG T-3’ and 5’-TCC CTC CCT AAA ACA ACT ACT ACC CA-3’ for unmethylated *MLH1* promoter. MSP PCR primer specificity was confirmed as they did not amplify non-bisulphite-treated genomic DNA templates, and the MSP products of several primary tumors have been confirmed by direct sequencing with BigDye v3.1 (Applied Biosystems), indicating that our MSP system is specific.

### Statistical analysis

The primary objective of this study was to identify distinct clinicopathologic features associated with MMR status and *KRAS* mutation. Logistic regression models were used to detect associations of these characteristics with each of the *KRAS* mutations and MMR status. Kruskal-Wallis and χ^2^ (or Fisher’s exact) tests were used to compare continuous and categorical variables, respectively. Univariate logistic regression models were used to further categorize and define the final covariables used for multivariable analysis. Statistical tests were two-sided, and *P* values of 0.05 were considered significant. For multiple comparisons, Bonferroni-adjusted *P* values were reported for the differences between *KRAS* mutation and MMR status (α = 0.05/6). Statistically significant characteristics based on univariate models were then included in multivariable models using stepwise and backwards model selection procedures. Odds ratios and their 95% confidence intervals were calculated. Statistics were carried out using SPSS software (version 16.0 of SPSS, Chicago, IL, USA).

## Results

**Fig 1:**
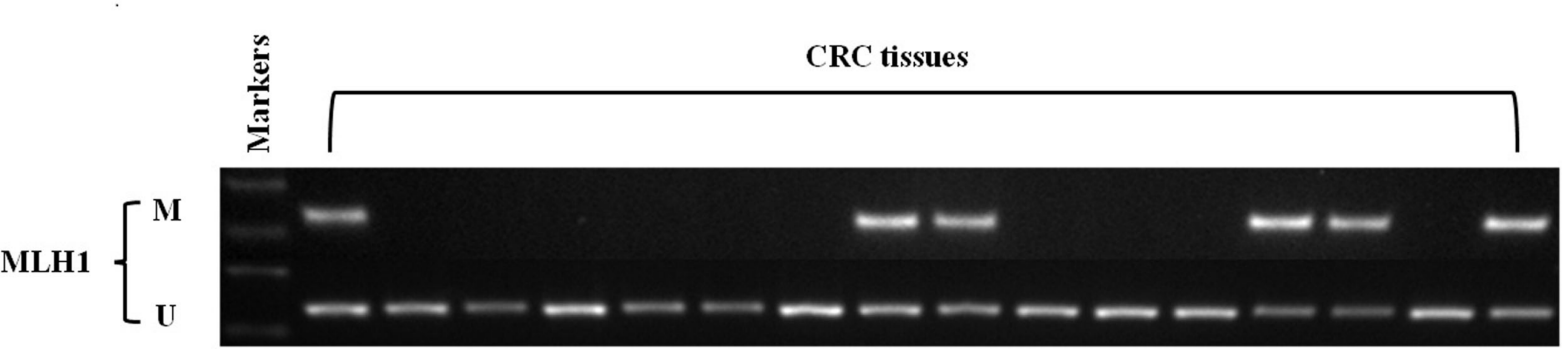
Representive MSP (Methylation-specific PCR) results of *MLH1* methylation in colorectal cancer with loss of MLH1/PMS2 protein expression.

A total of the 913 cases were evaluated by immunohistochemistry (IHC) for the presence or absence of MLH1, MSH2, MSH6 and PMS2 protein expression. Of the 69 cases (69/913, 7.5%) with dMMR, 49 had an absence of protein expression for MLH1/PMS2, 9 for MSH2/MSH6, 5 for MSH6 and 6 for PMS2 alone. In order to distinct Lynch syndrome-related CRC from sporadic MSI cancers, we performed the *MLH1* promoter methylation study. Among 49 cases with an absence of protein expression for MLH1/PMS2, 32.6% (16/49) cases had *MLH1* promoter methylation. Of them, *KRAS* mutations in *MLH1* methylated sporadic MSI tumors were 25% (4/16) and the mutation frequency was much lower than that of overall mutations, 36.9% (337/913) (Fig 1).

Patient and tumor characteristics with respect to the MMR status were shown in Table 1. The mean age at presentation for dMMR tumors was 53.3±12.7 years, which was younger than that of pMMR tumors (*P* = 0.003). Overall, tumors with dMMR were more frequently located on the proximal side of the colon (72.4% *vs* 18.9%, *P* < 0.0001) and were more likely to be poorly differentiated (34.8% *vs* 18.0%, *P* < 0.001), compared with pMMR tumors. In addition, tumors with dMMR were also significantly associated with mucinous histological subtype (63.8% *vs* 32.7%, *P* < 0.0001) and reduced lymph node metastasis (39.1% *vs* 54.6%, *P* = 0.01). There were no statistically significant differences in gender and pT stage.

**Table 1:** Distributions of clinicopathologic characteristics by MMR status.

Characterics	pMMR (n = 844)	dMMR (n = 69)	*P*-value
**Sex**			0.54
Male	494 (58.5%)	43 (62.3%)	
Female	350 (41.5%)	26 (37.7%)	
**Tumor Location**			<0.0001
Proximal colon	158 (18.9%)	42 (72.4%)	
Distal colon	259 (31.0%)	14 (24.1%)	
Rectum	419 (50.1%)	2 (3.4%)	
**pT stage**			0.06
pT1-2	117 (13.9%)	4 (5.8%)	
pT3-4	727 (86.1%)	65 (94.2%)	
**pN stage**			0.01
pN0	383 (45.4%)	42 (60.9%)	
pN1-2	461 (54.6%)	27 (39.1%)	
**Tumor Grade**			0.001
G1-2	692 (82.0%)	45 (65.2%)	
G3	152 (18.0%)	24 (34.8%)	
**Histological type**			<0.0001
Mucinous	276 (32.7%)	44 (63.8%)	
Non-mucinous	568 (67.3%)	25 (36.2%)	
**Disease stage**			0.02
I–II	374 (44.3%)	41 (59.4%)	
III–IV	470 (55.7%)	28 (40.6%)	
**Age, y**			0.003^†^
Mean (SD)	57.5±11.1	53.3±12.7	
Median	58.0	55.0	
Range	21.0–87.0	27.0–82.0	
**Age, y**			0.008
<50	197 (23.3%)	26 (37.7%)	
≥50	647 (76.7%)	43 (62.3%)	
**Tumor Size**			<0.0001
<6cm	658 (78.0%)	29 (42.0%)	
≥6cm	186 (21.0%)	40 (58.0%)	

Abbreviations: MMR = mismatch repair; SD = standard deviation.

^†^ Two-sided Kruskal Wallis test

Others are two-sided χ^2^ test.

Mutations in *KRAS* and *BRAF* ^*V600E*^ were mutually exclusive. There were 6 cases with dMMR tumors that harbored *BRAF* ^*V600E*^ mutations and these cases were excluded from the analysis. *KRAS* mutations in codons 12 and 13 were observed in 36.9% (337/913) of all tumors. A higher frequency of *KRAS* mutations were detected in dMMR tumors (27/69, 39.1%) compared with pMMR tumors (310/844, 36.7%), although this difference did not reach statistical significance. Of the 27 dMMR and *KRAS*-mutated tumors, 16 cases were defined with loss of MLH1/PMS2, 7 with MSH2/MSH6 and 4 with PMS2 alone.

Patient and tumor characteristics with respect to both the MMR and *KRAS* mutation status were summarized in Table 2. Of the 913 cases where both the MMR and *KRAS* mutation status were defined, 27 (2.9%) cases with dMMR were *KRAS* (+) and 42 (4.6%) were *KRAS* (-), whereas 310 (34.0%) cases with pMMR were *KRAS* (+) and 534 (58.5%) were *KRAS* (-). Among these four groups, significant differences were observed for gender {*KRAS* (+)/dMMR cases more likely to be male, *P* < 0.0001}, age {*KRAS* (+)/dMMR more likely to have a younger age at diagnosis of disease, *P* = 0.0001}, grade {*KRAS* (-)/dMMR more likely to have lower grade disease, *P* = 0.002}, tumor location {*KRAS* (+) and *KRAS* (-)/dMMR cases more likely to be located in the proximal colon, *P* < 0.0001}and lymph node metastasis {*KRAS* (+)/pMMR cases more likely to have higher pN stage, *P* = 0.004}. However, no differences were noted in the pT stage among these four groups.

**Table 2:** Clinicopathological characteristics by *KRAS* mutation status and MMR status.

Characteristics	pMMR Mutant *KRAS* (n = 310)	pMMR Wild-type *KRAS* (n = 534)	*P*-value	dMMR Mutant *KRAS* (n = 27)	dMMR Wild-type *KRAS* (n = 42)	*P*-value	*P*-value (Compared with 4 groups)
**Sex**			<0.0001			0.27	<0.0001
Male	152 (51.0%)	342 (64.0%)		19 (70.4%)	24 (57.1%)		
Female	158 (49.0%)	192 (36.0%)		8 (29.6%)	18 (42.9%)		
**Tumor Location**			0.004			0.51^§^	<0.0001
Proximal	60 (20.9%)	78 (14.7%)		16 (64.0%)	26 (66.7%)		
Distal	72 (25.1%)	187 (35.3%)		7 (28.0%)	7 (17.9%)		
Rectum	155 (54.0%)	264 (49.9%)		2 (8.0%)	6 (15.4%)		
**pT stage**			0.54			0.15^‡^	0.32^§^
pT1-2	40 (12.9%)	77 (14.4%)		6 (22.2%)	3 (7.1%)		
pT3-4	270 (87.1%)	457 (85.6%)		21 (77.8%)	39 (92.9%)		
**pN stage**			0.007			0.43	0.004
pN0	123 (39.7%)	263 (49.3%)		18 (66.7%)	24 (57.1%)		
pN1-2	187 (60.3%)	271 (50.7%)		9 (33.3%)	18 (42.9%)		
**Tumor Grade**			0.28			0.22	0.002
G1-2	260 (83.9%)	432 (80.9%)		20 (74.1%)	25 (59.5%)		
G3	50 (16.1%)	102 (19.1%)		7 (25.9%)	17 (40.5%)		
**Histological type**			0.007			0.36	<0.0001
Non-mucinous	191 (61.6%)	377 (70.6%)		8 (29.6%)	17 (40.5%)		
Mucinous	119 (38.4%)	157 (29.4%)		19 (70.4%)	25 (59.5%)		
**Disease stage**			0.03			0.63	0.01
I–II	122 (39.4%)	252 (47.2%)		17 (63.0%)	24 (57.1%)		
III–IV	188 (60.6%)	282 (52.8%)		10 (37.0%)	18 (42.9%)		
**Age, y**			0.07^†^			0.001^†^	0.001^†^
Mean (SD)	58.4 ± 10.8	56.9 ± 11.3		51.0 ± 12.2	54.8 ± 12.5		
Median	59.0	58.0		51.0	56.5		
Range	31.0–84.0	21.0–87.0		27.0–77.0	32.0–82.0		
**Age, y**			0.06			0.35	0.009
< 50	61 (19.7%)	135 (25.3%)		12 (44.4%)	14 (33.3%)		
≥ 50	249 (80.3%)	399 (74.7%)		15 (55.6%)	28 (66.7%)		
**Tumor Size**			0.61			0.50	<0.0001
< 6cm	245 (79.0%)	414 (77.5%)		10 (37.0%)	19 (45.2%)		
≥ 6cm	65 (21.0%)	120 (22.5%)		17 (63.0%)	23 (54.8%)		

Abbreviations: MMR = mismatch repair; SD = standard deviation.

^†^Two-sided Kruskal Wallis test

^‡^Two-sided χ^2^ test with continuity correction

^§^ Fischer’s exact test

Others are two-sided χ^2^ test

A *P* value for significance was adjusted for multiple hypothesis testing to *P* = 0.05/6 = 0.0083. Thus, a *P* value between 0.05 and 0.0083 should be regarded as of borderline significance.

When compared with those with *KRAS*(-)/pMMR tumors, patients with *KRAS*(+)/pMMR tumors were most likely to be female (49.0% *vs* 36.0%; OR = 1.85; 95% CI = 1.39 to 2.46; *P* < 0.0001), to be proximal located (45.5% *vs* 29.3%; OR = 2.00; 95% CI = 1.30 to 3.08; *P* = 0.002), to have a mucinous histology (38.4% *vs* 29.4%; OR = 1.50; 95% CI = 1.11 to 2.01; *P* = 0.007), and to have increased lymph node metastasis (60.3% *vs* 50.7%; OR = 1.48; 95% CI = 1.11 to 1.96; *P* = 0.007) (Table 3). To the contrary, compared with those with *KRAS*(-)/dMMR tumors, patients with *KRAS*(+)/dMMR tumors demonstrated no statistically significant differences in gender, tumor location, pT depth of invasion, lymph node metastasis, pTNM stage, and histologic grade. However, the mean age at presentation for *KRAS*(+)/dMMR tumors was 51.051.051.0 presentation for s,n, than that of *KRAS*(-)/dMMR tumors (*P* = 0.001).

**Table 3:** Univariate logistic regression model associations between *KRAS* mutation status and MMR status.

	pMMR-Mutant *KRAS*		dMMR-Mutant *KRAS*	
Characteristics	OR (95% CI)	*P*	OR (95% CI)	*P*
Female (referent: male)	1.85 (1.39 to 2.46)	<0.0001	0.56 (0.20 to 1.57)	0.37
Proximal (referent: distal)	2.00 (1.30 to 3.08)	0.002	1.63 (0.48 to 5.50)	0.43
Proximal (referent: Rectum)	1.31 (0.89 to 1.93)	0.17	0.54 (0.10 to 3.02)	0.76
pT 3–4 (referent: pT 1–2)	1.14 (0.75 to 1.72)	0.54	0.27 (0.06 to 1.19)	0.15
pN 1–2 (referent: pN 0)	1.48 (1.11 to 1.96)	0.007	0.67 (0.24 to 1.82)	0.43
Low grade (referent: moderate to high grade)	0.81 (0.56 to 1.18)	0.28	0.51 (0.18 to 1.48)	0.22
Mucinous (referent: non-mucinous)	1.50 (1.11 to 2.01)	0.007	1.61 (0.58 to 4.53)	0.36
pTNM III–IV (referent: pTNM I–II)	1.38 (1.04 to 1.83)	0.03	0.78 (0.29 to 2.11)	0.63
Age ≥ 50 years (referent: < 50 years)	1.38 (0.98 to 1.94)	0.06	0.63 (0.23 to 1.69)	0.35
Tumor size < 6cm (referent: ≥ 6cm)	1.09 (0.78 to 1.54)	0.61	0.71 (0.26 to 1.92)	0.50

CI = confidence interval; dMMR = deficient mismatch repair; pMMR = proficient mismatch repair; OR = odds ratio.

^‡^ = Two-sided χ^2^ test with continuity correction

^§^ Fischer’s exact test

Others are two-sided χ^2^ test.

A *P* value for significance was adjusted for multiple hypothesis testing to P = 0.05/6 = 0.0083. Thus, a *P* value between 0.05 and 0.0083 should be regarded as of borderline significance.

In the analysis using multivariable logistic regression models, we reviewed clinicopathologic characteristics in Table 4. As shown multivariably, tumors with *KRAS*(+)/pMMR were statistically associated with proximal location, mucinous histology and increased lymph node metastasis.

**Table 4:** Multivariate logistic regression model associations between patient, tumor and *KRAS* or *BRAF* ^*V600E*^ mutation status.

Characteristics	pMMR-Mutant *KRAS*	
	OR (95% CI)	*P*
Female (referent: male)	2.73 (1.69 to 4.85)	0.001
Proximal (referent: distal)	2.17 (1.39 to 3.33)	0.001
pN 1–2 (referent: pN 0)	4.95 (2.84 to 6.38)	0.003
Mucinous (referent: non-mucinous)	3.44 (1.11 to 7.47)	0.02
pTNM III–IV (referent: pTNM I–II)	0.26 (0.03 to 2.02)	0.19

CI = confidence interval; pMMR = proficient mismatch repair; OR = odds ratio.

## Discussion

Defining tumor subtypes of CRC based on pathway-driven alterations has the potential to improve prognostication and guide targeted therapy. Distinct clinical and pathological features of CRC with different MMR status have long been identified [6,26,27,28]. In this study, we demonstrated molecular and clinicopathological features of pMMR and dMMR tumors stratified by *KRAS* mutation status in a large cohort of consecutive Chinese CRC patients. Proficient MMR tumors that were nonmutated for *KRAS* and *BRAF* ^*V600E*^ were the most prevalent subtype and represented 58.5% (534/913) of our study cohort. Compared with this subtype, patients with *KRAS*(+)/pMMR tumors were more common in the proximal colon and to have a mucinous histology. Most importantly, patients with *KRAS*(+)/pMMR tumors showed increased lymph node metastasis among four subtypes and may have worse survival rate.

Consistent with the previous findings, our data suggest that tumors with dMMR status often exhibit poor differentiation, mucinous cell type, proximal location and reduced lymph node metastasis. In addition to its role in identifying unique pathological features of CRC, dMMR status has also been used as a prognostic marker and the guidance for Fluorouracil-based adjuvant chemotherapy [8]. Recent evidence indicated that CRC could be further classified into five prespecified subtypes using a biomarker combination of *KRAS* and *BRAF* ^*V600E*^ mutations, MMR status and *MLH1* methylation with statistically significant differences in clinicopathologic features and patient survival rates [29]. Thus, a biomarker-based classifier provides important prognostic information in CRC with implications for patient management. Evidence from other reports supported the idea that *KRAS* mutation and MMR status are genetic markers that arise early and remain biologically relevant throughout all stages of tumor progression [30,31]. In addition, *KRAS* mutations found in primary tumors are preserved in recurrences and metastases. Consequently, we evaluated the prespecified tumor subtypes with respect to clinicopathologic features in biomarker combinations of *KRAS* mutations and MMR status.

Deficient MMR cancers typically originate in the proximal colon [15]. As expected, the vast majority of dMMR tumors in this study (72.4%) were from the proximal colon and this distribution was not influenced by *KRAS* mutations. Although most of the pMMR tumors were not likely to be proximal located, it is interesting that when pMMR tumors stratified by *KRAS* mutations, *KRAS* mutant tumors (20.9% proximal) were more likely to be proximal compared to *KRAS*(-)/pMMR tumors (14.7% proximal). Traditionally, colon cancers developed in the proximal bowel often created an environment in which CIMP (CpG Island Methylator Phenotype) is more likely to arise, and this synergizes with *BRAF* mutation to allow progression of serrated polyps [32]. However, recent evidence suggested that *KRAS* mutations could also be found in CIMP high and CIMP low tumors which were often located in the proximal colon [33]. This indicated that there were more comprehensive mechanisms underlying the location of colon cancer and the mutational profiles. Mucinous carcinoma is diagnosed when at least 50% of the tumor comprises secretory mucin and is often associated with dMMR status and serrated adenocarcinoma [34]. This is consistent with our finding that dMMR tumors demonstrated more mucinous differentiation than pMMR tumors (63.8% *vs* 32.7%). However, when stratified by *KRAS* mutation status, we observed that pMMR tumors with mutant *KRAS* phenotype exhibited more mucinous differentiation than wild type *KRAS* subtype (38.4% *vs* 29.4%). This is largely because *KRAS* mutation is not only linked to conventional adenomas but also associated with serrated adenomas in the development of colorectal cancer [35].

A significant association was found between the presence of lymph node metastases and pMMR tumors stratified by *KRAS* mutation status. Our findings revealed that pMMR tumors with *KRAS* mutation demonstrated more positive lymph nodes and pTNM III-IV stage of disease than tumors with *KRAS*(-)/pMMR status. This is consistent with findings from a smaller report, which demonstrated that the frequency of *KRAS* mutations was higher in pMMR lymph node positive tumors as compared to pMMR lymph node negative tumors [36,37]. Our results indicate that the majority of pMMR tumors needed *KRAS* mutation to be able to metastasize and this activation was crucial for neoplastic cells to acquire invasive potential. Mutations in *KRAS* oncogene lead to alterations in encoded amino acids adjacent to the GTP binding pocket and reduced the GTPase activity of *KRAS* protein after guanine nucleotide activating protein (GAP) binding [38]. Both *in vitro* and *in vivo* experimental models, transfection of mutated, constitutively active forms of *KRAS* oncogene into previously noncancerous cells can lead to invasive and metastatic phenotypes. Ectopic expression of active *KRAS* in the murine NIH 3T3 fibroblast cell line resulted in increased invasion and acquisition of metastatic properties [39]. Using tail vein injection of transformed cells, *in vivo* models were observed by liver and lung metastasis [40]. In addition to the evidence obtained from cell and animal experiments, clinical studies have also displayed significant lymph node metastasis in *KRAS*(+)/pMMR tumors [36,41]. Gene expression profiling reveals that genes involoving epithelial mesenchymal transition and matrix remodeling that can facilitate tumor invasion and metastasis are up-regulated in mutant *KRAS*-pMMR tumors [42]. Consequently, *KRAS* oncogenic activation was shown to be an important mediator of tumor cell invasion and metastasis in pMMR tumors.

The frequency of *KRAS* mutations in Lynch syndrome-related CRC and sporadic CRC is almost the same. However, *KRAS* mutations are significantly more frequent in Lynch syndrome-related CRC than that in sporadic MSI-H CRCs [37,43]. Lynch syndrome-related CRC tend to be early-onset and proximal location. So this may explain the younger age and proximal location observed in *KRAS*+/MSI tumors. Despite these positive findings, our study has some limitations. First, because this is a retrospectively study, it is hard to collect the blood or saliva sample from patients to detect germline mutations to further distinguish the Lynch syndrome-related CRC from sporadic cancer. So we could not calculate the precise frequency of *KRAS* mutations in hereditary CRCs, however, it is sure that the Lynch syndrome-related CRC in our study showed preferentially *KRAS* mutations. Second, we did not examine other less common mutations in *KRAS* codons 61, 117 and 146, which also contributed to the oncogenic transformation of tumor cells.

This study suggests that specific epidemiologic and clinicopathologic characteristics are associated with MMR status stratified by *KRAS* mutation in CRC. Knowledge of MMR and *KRAS* mutation status may enhance molecular pathologic staging of CRC patients and metastatic progression can also be estimated based on the combination of these biomarkers. Validation of additional genetic biomarkers will help to refine management decisions for individual patients based on tumor biology. Importantly, this may also aid the development of novel therapeutic targets to aid treatment of these aggressive cancers.

## References

[1] Fearon ER (2011) Molecular genetics of colorectal cancer. Annu Rev Pathol 6: 479–507. 10.1146/annurev-pathol-011110-130235 21090969

[2] Grady WM, Carethers JM (2008) Genomic and epigenetic instability in colorectal cancer pathogenesis. Gastroenterology 135: 1079–1099. 10.1053/j.gastro.2008.07.076 18773902 PMC2866182

[3] Jass JR (2007) Classification of colorectal cancer based on correlation of clinical, morphological and molecular features. Histopathology 50: 113–130. 17204026 10.1111/j.1365-2559.2006.02549.x

[4] Siegel R, Desantis C, Jemal A (2014) Colorectal cancer statistics, 2014. CA Cancer J Clin 64: 104–117. 10.3322/caac.21220 24639052

[5] Sinicrope FA, Sargent DJ (2012) Molecular pathways: microsatellite instability in colorectal cancer: prognostic, predictive, and therapeutic implications. Clin Cancer Res 18: 1506–1512. 10.1158/1078-0432.CCR-11-1469 22302899 PMC3306518

[6] Ribic CM, Sargent DJ, Moore MJ, Thibodeau SN, French AJ, Goldberg RM, et al (2003) Tumor microsatellite-instability status as a predictor of benefit from fluorouracil-based adjuvant chemotherapy for colon cancer. N Engl J Med 349: 247–257. 12867608 10.1056/NEJMoa022289 PMC3584639

[7] Sinicrope FA, Mahoney MR, Smyrk TC, Thibodeau SN, Warren RS, Bertagnolli MM, et al (2013) Prognostic impact of deficient DNA mismatch repair in patients with stage III colon cancer from a randomized trial of FOLFOX-based adjuvant chemotherapy. J Clin Oncol 31: 3664–3672. 10.1200/JCO.2013.48.9591 24019539 PMC3789216

[8] Sinicrope FA, Foster NR, Thibodeau SN, Marsoni S, Monges G, Labianca R, et al (2011) DNA mismatch repair status and colon cancer recurrence and survival in clinical trials of 5-fluorouracil-based adjuvant therapy. J Natl Cancer Inst 103: 863–875. 10.1093/jnci/djr153 21597022 PMC3110173

[9] Popat S, Hubner R, Houlston RS (2005) Systematic review of microsatellite instability and colorectal cancer prognosis. J Clin Oncol 23: 609–618. 15659508 10.1200/JCO.2005.01.086

[10] Umar A, Risinger JI, Hawk ET, Barrett JC (2004) Testing guidelines for hereditary non-polyposis colorectal cancer. Nat Rev Cancer 4: 153–158. 14964310 10.1038/nrc1278

[11] Salovaara R, Loukola A, Kristo P, Kaariainen H, Ahtola H, Eskelinen M, et al (2000) Population-based molecular detection of hereditary nonpolyposis colorectal cancer. J Clin Oncol 18: 2193–2200. 10829038 10.1200/JCO.2000.18.11.2193

[12] Davies H, Bignell GR, Cox C, Stephens P, Edkins S, Clegg S, et al (2002) Mutations of the BRAF gene in human cancer. Nature 417: 949–954. 12068308 10.1038/nature00766

[13] Wang L, Cunningham JM, Winters JL, Guenther JC, French AJ, Boardman LA, et al (2003) BRAF mutations in colon cancer are not likely attributable to defective DNA mismatch repair. Cancer Res 63: 5209–5212. 14500346

[14] Nagasaka T, Koi M, Kloor M, Gebert J, Vilkin A, Nishida N, et al (2008) Mutations in both KRAS and BRAF may contribute to the methylator phenotype in colon cancer. Gastroenterology 134: 1950–1960, 1960 e1951 10.1053/j.gastro.2008.02.094 18435933 PMC2543132

[15] Jass JR, Do KA, Simms LA, Iino H, Wynter C, Pillay SP, et al (1998) Morphology of sporadic colorectal cancer with DNA replication errors. Gut 42: 673–679. 9659163 10.1136/gut.42.5.673 PMC1727100

[16] Perkins G, Pilati C, Blons H, Laurent-Puig P (2014) Beyond KRAS status and response to anti-EGFR therapy in metastatic colorectal cancer. Pharmacogenomics 15: 1043–1052. 10.2217/pgs.14.66 24956256

[17] Lengauer C, Kinzler KW, Vogelstein B (1997) Genetic instability in colorectal cancers. Nature 386: 623–627. 9121588 10.1038/386623a0

[18] Karapetis CS, Khambata-Ford S, Jonker DJ, O'Callaghan CJ, Tu D, Tebbutt NC, et al (2008) K-ras mutations and benefit from cetuximab in advanced colorectal cancer. N Engl J Med 359: 1757–1765. 10.1056/NEJMoa0804385 18946061

[19] Normanno N, Tejpar S, Morgillo F, De Luca A, Van Cutsem E, Ciardiello F. (2009) Implications for KRAS status and EGFR-targeted therapies in metastatic CRC. Nat Rev Clin Oncol 6: 519–527. 10.1038/nrclinonc.2009.111 19636327

[20] Moosmann N, Heinemann V (2007) Cetuximab in the treatment of metastatic colorectal cancer. Expert Opin Biol Ther 7: 243–256. 17250462 10.1517/14712598.7.2.243

[21] Samowitz WS, Curtin K, Schaffer D, Robertson M, Leppert M, Slattery ML. (2000) Relationship of Ki-ras mutations in colon cancers to tumor location, stage, and survival: a population-based study. Cancer Epidemiol Biomarkers Prev 9: 1193–1197. 11097226

[22] Rosty C, Young JP, Walsh MD, Clendenning M, Walters RJ, Pearson S, et al (2013) Colorectal carcinomas with KRAS mutation are associated with distinctive morphological and molecular features. Mod Pathol 26: 825–834. 10.1038/modpathol.2012.240 23348904

[23] Zlobec I, Kovac M, Erzberger P, Molinari F, Bihl MP, Rufle A, et al (2010) Combined analysis of specific KRAS mutation, BRAF and microsatellite instability identifies prognostic subgroups of sporadic and hereditary colorectal cancer. Int J Cancer 127: 2569–2575. 10.1002/ijc.25265 20162668

[24] Yoon HH, Tougeron D, Shi Q, Alberts SR, Mahoney MR, Nelson GD, et al (2014) KRAS codon 12 and 13 mutations in relation to disease-free survival in BRAF-wild-type stage III colon cancers from an adjuvant chemotherapy trial (N0147 alliance). Clin Cancer Res 20: 3033–3043. 10.1158/1078-0432.CCR-13-3140 24687927 PMC4040326

[25] Zhao H, Li Q, Wang J, Su X, Ng KM, Qiu T, et al (2012) Frequent epigenetic silencing of the folate-metabolising gene cystathionine-beta-synthase in gastrointestinal cancer. PLoS One 7: e49683 10.1371/journal.pone.0049683 23152928 PMC3496708

[26] Campbell PT, Jacobs ET, Ulrich CM, Figueiredo JC, Poynter JN, McLaughlin JR, et al (2010) Case-control study of overweight, obesity, and colorectal cancer risk, overall and by tumor microsatellite instability status. J Natl Cancer Inst 102: 391–400. 10.1093/jnci/djq011 20208017 PMC2841037

[27] Sargent DJ, Marsoni S, Monges G, Thibodeau SN, Labianca R, Hamilton SR, et al (2010) Defective mismatch repair as a predictive marker for lack of efficacy of fluorouracil-based adjuvant therapy in colon cancer. J Clin Oncol 28: 3219–3226. 10.1200/JCO.2009.27.1825 20498393 PMC2903323

[28] Sinicrope F, Foster NR, Sargent DJ, Thibodeau SN, Smyrk TC, O'Connell MJ, et al (2010) Model-based prediction of defective DNA mismatch repair using clinicopathological variables in sporadic colon cancer patients. Cancer 116: 1691–1698. 10.1002/cncr.24913 20186699 PMC2855300

[29] Sinicrope FA, Shi Q, Smyrk TC, Thibodeau SN, Dienstmann R, Guinney J, et al (2015) Molecular Markers Identify Subtypes of Stage III Colon Cancer Associated With Patient Outcomes. Gastroenterology 148: 88–99. 10.1053/j.gastro.2014.09.041 25305506 PMC4274188

[30] Nash GM, Gimbel M, Cohen AM, Zeng ZS, Ndubuisi MI, Nathanson DR, et al (2010) KRAS mutation and microsatellite instability: two genetic markers of early tumor development that influence the prognosis of colorectal cancer. Ann Surg Oncol 17: 416–424. 10.1245/s10434-009-0713-0 19813061 PMC4380015

[31] Asaka S, Arai Y, Nishimura Y, Yamaguchi K, Ishikubo T, Yatsuoka T, et al (2009) Microsatellite instability-low colorectal cancer acquires a KRAS mutation during the progression from Dukes' A to Dukes' B. Carcinogenesis 30: 494–499. 10.1093/carcin/bgp017 19147861

[32] French AJ, Sargent DJ, Burgart LJ, Foster NR, Kabat BF, Goldberg R, et al (2008) Prognostic significance of defective mismatch repair and BRAF V600E in patients with colon cancer. Clin Cancer Res 14: 3408–3415. 10.1158/1078-0432.CCR-07-1489 18519771 PMC2674786

[33] The Cancer Genome Altas Network. (2012) Comprehensive molecular characterization of human colon and rectal cancer. Nature 487: 330–337. 10.1038/nature11252 22810696 PMC3401966

[34] Whitehall VL, Wynter CV, Walsh MD, Simms LA, Purdie D, Pandeya N, et al (2002) Morphological and molecular heterogeneity within nonmicrosatellite instability-high colorectal cancer. Cancer Res 62: 6011–6014. 12414620

[35] Ogino S, Chan AT, Fuchs CS, Giovannucci E (2011) Molecular pathological epidemiology of colorectal neoplasia: an emerging transdisciplinary and interdisciplinary field. Gut 60: 397–411. 10.1136/gut.2010.217182 21036793 PMC3040598

[36] Oliveira C, Velho S, Moutinho C, Ferreira A, Preto A, Domingo E, et al (2007) KRAS and BRAF oncogenic mutations in MSS colorectal carcinoma progression. Oncogene 26: 158–163. 16953233 10.1038/sj.onc.1209758

[37] Oliveira C, Westra JL, Arango D, Ollikainen M, Domingo E, Ferreira A, et al (2004) Distinct patterns of KRAS mutations in colorectal carcinomas according to germline mismatch repair defects and hMLH1 methylation status. Hum Mol Genet 13: 2303–2311. 15294875 10.1093/hmg/ddh238

[38] Malumbres M, Barbacid M (2003) RAS oncogenes: the first 30 years. Nat Rev Cancer 3: 459–465. 12778136 10.1038/nrc1097

[39] Campbell PM, Der CJ (2004) Oncogenic Ras and its role in tumor cell invasion and metastasis. Semin Cancer Biol 14: 105–114. 15018894 10.1016/j.semcancer.2003.09.015

[40] Al-Mulla F, MacKenzie EM (2001) Differences in in vitro invasive capacity induced by differences in Ki-Ras protein mutations. J Pathol 195: 549–556. 11745690 10.1002/path.995

[41] Roth AD, Tejpar S, Delorenzi M, Yan P, Fiocca R, Klingbiel D, et al (2010) Prognostic role of KRAS and BRAF in stage II and III resected colon cancer: results of the translational study on the PETACC-3, EORTC 40993, SAKK 60–00 trial. J Clin Oncol 28: 466–474. 10.1200/JCO.2009.23.3452 20008640

[42] Marisa L, de Reynies A, Duval A, Selves J, Gaub MP, Vescovo L, et al (2013) Gene expression classification of colon cancer into molecular subtypes: characterization, validation, and prognostic value. PLoS Med 10: e1001453 10.1371/journal.pmed.1001453 23700391 PMC3660251

[43] Garre P, Martin L, Bando I, Tosar A, Llovet P, Sanz J, et al (2014) Cancer risk and overall survival in mismatch repair proficient hereditary non-polyposis colorectal cancer, Lynch syndrome and sporadic colorectal cancer. Fam Cancer 13: 109–119. 10.1007/s10689-013-9683-2 24061861

